# Targeted Long-Read Sequencing of a Locus Under Long-Term Balancing Selection in *Capsella*

**DOI:** 10.1534/g3.117.300467

**Published:** 2018-02-20

**Authors:** Jörg A. Bachmann, Andrew Tedder, Benjamin Laenen, Kim A. Steige, Tanja Slotte

**Affiliations:** Department of Ecology, Environment and Plant Sciences, Science for Life Laboratory, Stockholm University, Sweden

**Keywords:** single-molecule real-time sequencing, bacterial artificial chromosomes, sequencing errors, assembly, self-incompatibility locus, *Capsella*, Brassicaceae

## Abstract

Rapid advances in short-read DNA sequencing technologies have revolutionized population genomic studies, but there are genomic regions where this technology reaches its limits. Limitations mostly arise due to the difficulties in assembly or alignment to genomic regions of high sequence divergence and high repeat content, which are typical characteristics for loci under strong long-term balancing selection. Studying genetic diversity at such loci therefore remains challenging. Here, we investigate the feasibility and error rates associated with targeted long-read sequencing of a locus under balancing selection. For this purpose, we generated bacterial artificial chromosomes (BACs) containing the Brassicaceae *S*-locus, a region under strong negative frequency-dependent selection which has previously proven difficult to assemble in its entirety using short reads. We sequence *S*-locus BACs with single-molecule long-read sequencing technology and conduct *de novo* assembly of these *S*-locus haplotypes. By comparing repeated assemblies resulting from independent long-read sequencing runs on the same BAC clone we do not detect any structural errors, suggesting that reliable assemblies are generated, but we estimate an indel error rate of 5.7×10^−5^. A similar error rate was estimated based on comparison of Illumina short-read sequences and BAC assemblies. Our results show that, until *de novo* assembly of multiple individuals using long-read sequencing becomes feasible, targeted long-read sequencing of loci under balancing selection is a viable option with low error rates for single nucleotide polymorphisms or structural variation. We further find that short-read sequencing is a valuable complement, allowing correction of the relatively high rate of indel errors that result from this approach.

DNA sequencing has come a long way since Sanger’s “chain termination” technique first improved our ability to sequence DNA (Sanger and Nicklen 1977). In the last decade, major breakthroughs in massively parallel sequencing have unfolded whole new generations of technologies, currently allowing us to reliably uncover vast amounts of genomic information (Heather and Chain 2016). However, despite recent advances in high-throughput (short-read) sequencing technologies (Reuter *et al.* 2015), some genetic regions remain difficult to study using short read data due to their size and complex architecture (Mardis 2017). Notably, loci under long-term balancing selection, such as the Major-Histocompatibility (*MHC*) locus (Hedrick 1999) or plant self-incompatibility (*S*) loci as in the Brassicaceae *S*-locus, have a large number of highly divergent alleles maintained by negative frequency-dependent selection over long periods of time (Wright 1939; Vekemans and Slatkin 1994; Charlesworth *et al.* 2000; Castric and Vekemans 2007). Their genetic architecture is affected by long-term balancing selection that promotes the emergence and co-existence of numerous differentiated alleles (Llaurens *et al.* 2017). In such regions, high repeat content, diversity, and rearrangements makes assembly and especially re-sequencing approaches based on mapping short reads to a reference genome, difficult (Mardis 2017).

Previously, studies on genetic diversity at the *S*-locus would rely on polymerase chain-reaction (PCR) amplification of specific regions of interest (often the *S*-locus receptor kinase gene *SRK*) in combination with either Sanger sequencing (Shiba 2001; Kusaba *et al.* 2001; Miege *et al.* 2001; Schierup *et al.* 2001; Charlesworth, *et al.* 2003a; 2003b; Nasrallah *et al.* 2004; Bechsgaard *et al.* 2006; Kamau *et al.* 2007; Castric *et al.* 2010; Tsuchimatsu *et al.* 2012; Leducq *et al.* 2014) or short-read sequencing (Guo *et al.* 2009; Jørgensen *et al.* 2012). The two major caveats of this approach are; it is not always applicable to use (general) PCR primers for very divergent alleles, and regulatory regions in intergenic regions of high complexity are not resolved. For this reason, researchers have resorted to targeted sequencing of the entire *S*-locus region using massively parallel sequencing of bacterial artificial chromosomes (BACs) containing the *S*-locus (Guo *et al.* 2011; Goubet *et al.* 2012; Durand *et al.* 2014; Novikova *et al.* 2017; Tsuchimatsu *et al.* 2017). However, due to the high repeat content of the *S*-locus, next-generation sequencing of and assembly of *S*-locus BACs with short- or medium-length reads resulted in several contigs, thus requiring additional PCR-based testing to elucidate gene order and orientation (Goubet *et al.* 2012).

In contrast to short-read sequencing technologies, SMRT sequencing should provide a better basis for reliably assembling repetitive regions, due to mean read lengths >20 kb and maximum reads >60 kb (Rhoads and Au 2015). Eventually, *de novo* assembly of whole genomes for each individual would be the goal for the study of evolution of loci under long-term balancing selection. However, the high costs per base of SMRT sequencing currently limit the feasibility of this approach, especially for studying population genetic variation at loci under balancing selection. Here, we therefore investigate the utility of targeted sequencing of two different Brassicaceae *S*-locus sequences in two BACs using SMRT sequencing, with a focus on quantifying assembly errors, single nucleotide polymorphism (SNP) errors and indel errors. By comparison of two assemblies of independent SMRT sequencing one of the two *S*-locus BACs, we find that this approach is efficient and highly accurate with regard to structural errors and single nucleotide polymorphisms. Mapping short-read data to the an SMRT assembly of the second *S*-locus BAC for error correction, we find that correction for indel errors is necessary, especially for studies aiming to identify functional polymorphisms. This method can thus be valuable for a wide range of genomic studies of complex genomic regions, where reference-based approaches for studying genetic variation are not feasible.

## Materials and Methods

### Plant material

We surface-sterilized seeds of four accessions of the self-incompatible crucifer *Capsella grandiflora* (from Epiros, Zagori, Greece) with 10% bleach and 70% ethanol. Seeds were stratified at 2-4° in the dark on plates with 0.8% agar and half-strength MS medium (Murashige and Skoog basal salt mixture, Sigma-Aldrich Co. MI, USA). After two weeks, we moved the plates to climate controlled growth chambers (16 h light at 20° / 8 h dark at 18°, 70% max. humidity, 122 uE light intensity) to allow the seeds to germinate. After approximately 1 week, we transplanted seedlings to pots with soil in the climate-controlled chambers. We kept the plants under dark conditions for 4 days prior to sampling young leaves for BAC library construction.

### BAC library construction and screening

To sequence full-length *S*-locus haplotypes, we followed a strategy similar to Goubet *et al.* (2012) based on BAC libraries. High molecular weight DNA was extracted from 10 g of young leaves per library, and we pooled leaves from two individuals per library. The DNA was digested with *Hin*dIII and ligated to pCC1BAC cloning vector (Epicentre, an Illumina company, WI, USA), after several size selection steps. BAC libraries were screened for flanking regions of the *S*-locus by hybridization with DNA probes and PCR amplification with specific primers and further selected based on mean insert size. Clones of colonies that tested positive for *U-box* and *ARK3* flanking genes of the *S*-locus were selected for sequencing. All BAC library production and screening was performed by the French Plant Genomic Resource Centre (CNRGV) at INRA.

### Sequencing

We conducted SMRT sequencing (Pacific Biosciences of California, CA, USA) of two different Brassicaceae *S*-locus sequences in two BAC clones at the Uppsala Genome Center, National Genomics Infrastructure Sweden. DNA fragments over 10 kbp were selected using BluePippin Size selection (Sage Science, MA, USA) and the SMRTbell Template Prep Kit 1.0 (Pacific Biosciences of California, CA, USA) was used for library preparation, with an insert size of 500 bp to 20 kb. SMRT sequencing was done on the RSII system, using P5-C3 chemistry.

To assess sequencing and assembly errors, we generated two independent libraries of one BAC clone (CgrS-BAC1), which was then subjected to independent SMRT sequencing and assembly, whereas the second BAC, (CgrS-BAC2), was sequenced once with SMRT sequencing. To assess indel errors, we also generated short-read sequencing (MiSeq, Illumina, Inc., San Diego, USA) data for the second *S*-locus BAC (CgrS-BAC2). The sequencing library (TruSeq PCRfree DNA sample preparation kit, Illumina, Inc., CA, USA) was prepared from 1 μg of DNA, following the manufacturers’ guidelines. We generated 1.1 million paired-end 250 bp reads on the MiSeq using v2 sequencing chemistry (Illumina, Inc., CA, USA).

### Bioinformatic data processing and assembly

We assembled raw SMRT reads from each BAC clone using the Hierarchical Genome Assembly Process (HGAP.3) (Chin *et al.* 2013) with default settings. The pipeline generates a *de novo* assembly with Celera Assembler 8.3rc2 (Myers *et al.* 2000) and includes a consensus polishing step using the Quiver algorithm (Pacific Biosciences of California, Inc., CA, USA). Per sequenced BAC clone, this process yielded a large assembled fragment (contig) containing the region of interest (*S*-locus), as well as several contigs containing *E. coli* sequences. As HGAP.3 does not split reads, assembling a circular molecule results in overlapping ends of reduced coverage, and we therefore conducted circularisation and removed overlapping ends using minimus2 v3.1.0 of the AMOS suite (Treangen *et al.* 2011) This was followed by another Quiver polishing step (Chin *et al.* 2013) to improve the quality in the region that was formerly split between the two ends of the sequence, and finally trimming of the vector sequence.

We quality filtered and trimmed raw reads from Illumina MiSeq sequencing to remove adapters using cutadapt v1.3 (Martin 2011) which identified the most likely used adapters. Subsequently, we trimmed all adapters as well as low-quality reads with Trimmomatic v0.36 (Bolger *et al.* 2014).

### Error estimation and correction

To estimate assembly and sequencing error rates, we compared the *S*-locus contigs from independent sequencing and assembly of CgrS-BAC1. We generated a pairwise alignment of the two *S*-locus assemblies using Mafft v7.310 (Katoh *et al.* 2002) and assessed the total number of assembly errors (*i.e.*, structural differences between the assemblies), and the numbers and base-pair locations of indels and SNPs, that represent sequencing errors.

To generate an additional estimate of sequencing error rates, we used Illumina MiSeq data for CgrS-BAC2. We mapped short reads to the polished assembly of the BAC clone using bwa-mem v 0.7.8 (Li and Durbin 2009). Finally, we estimated indel error rates and corrected these indel errors using pacbio-util, based on the consensus of the mapped Illumina reads (https://github.com/douglasgscofield/PacBio-utilities). Indel error rate was calculated as number of insertions and deletions, divided by assembly length to get a per base-pair error rate.

### Annotation and comparison of SRK sequences

To assess the utility of using SMRT sequencing of BAC clones to reconstruct complex loci, we extracted *SRK* exon 1 sequences from the *S*-haplotype assemblies by searching for BLAST hits to general *SRK* exon 1 forward (*SLGF*) and reverse (*SLGR*) primers (Charlesworth *et al.* 2000), extracting either the sequence between the two primer sites, or, if only one primer site was found, each 1kb sequence up- and downstream of the primer site. We then selected candidate sequences based on strong sequence similarity to known *SRK* exon 1 sequences or conversely rejected them based on stronger sequence homology to known *ARK3* (*Aly8*) sequences using BLAST (v2.5.0+). Exact parameters for sequence homology varied between candidate sequences due to high divergence in *SRK* alleles, but were always above 90%.

In order to characterize the relationship between our *Capsella SRK*-like sequences, and known *SRK* and *ARK3* alleles, we bulk downloaded *722* publicly available Brassicaceae *SRK* and *ARK3* sequences of >500 bp length from GenBank (Table S1) and retained only those under 2000 bp of length. Duplicates were removed using dedupe.sh from BBMAP v34.56 (Joint Genome Institute), and we made an initial alignment between our *SRK* sequences and the publically available *SRK* and *ARK3* using MAFFT v7.245 with the E-INS-I algorithm (Katoh *et al.* 2002), which is suitable for sequences containing large unalignable gaps. Due to the sequence diversity present in *SRK* exon 1, it was necessary for us to manually edit the alignment in Seaview v4.6 (Gouy *et al.* 2010) to correct alignment errors. To visualize the phylogenetic relationship between our *SRK* sequences and those previously sequenced, we constructed a phylogenetic tree using RaXMl v8.2.3 (Stamatakis 2014), generating a neighbor-joining tree with the GTRGAMMA model, and 1000 bootstrap replicates. The tree was visualized using FigTree v1.4.2 (http://tree.bio.ed.ac.uk/software/figtree/).

To assess whether we had successfully sequenced the entire *S*-locus, we annotated our *S*-locus assemblies with Augustus v3.2.3 (Stanke *et al.* 2004) and RepeatMasker v4.0.7 (http://www.repeatmasker.org) via Maker v2.31.9 (Holt and Yandell 2011), with *Arabidopsis thaliana* as a model prediction species and protein homology data for *B120*, *ARK3*, *SRK*, *U-box*, *B70*, *DYT1*, *SBT3* and *AT4G21323* from *Arabidopsis lyrata* and *A. halleri*. Annotation of the highly variable *S*-locus gene *SCR* was unsuccessful with a homology search to existing *SCR* alleles. Using a sliding window approach in open reading frames, we searched for conserved patterns of 8 cysteine residues to find *SCR* exon 2. The resultant *gff* files were concatenated, and the annotation visualized using R v3.3.1 (R Development Core Team 2008).

### Sequence conservation

To assess patterns of sequence conservation across the entire *S*-locus region between *ARK3* and *U-box*, we first extracted a larger region between B120 and *AT4G21323* as described above. *S*-locus sequences were then aligned using LASTZ v1.03.54 (Harris 2007) and the resultant “axt” files were converted to fasta format using axt2maf and maf2fasta, respectively. Pairwise sequence conservation, as the proportion of conserved bases per 250 bp sliding-window, was then calculated with a python script, and visualized using R v3.3.1 (R Development Core Team 2008) (https://gitlab.com/slottelab/Sequence_conservation).

### Data availability

The sequences of CgrS-BAC1 and CgrS-BAC2 we generated in this study have been uploaded to ENA at EBI with project id: PRJEB24927. Table S1 contains Genbank accession numbers of *SRK* sequences used in this study.

## Results and Discussion

### Sequencing and Assembly

SMRT sequencing of two BAC clones corresponding to two different *S*-haplotypes resulted in an N50 read length of 19,187 to 28,120 bp (Table 1). For additional short-read data for one of the BACs that was assembled based on long-read data, CgrS-BAC2, we obtained a total of 482.1 Mbp of Illumina MiSeq paired-end data (250 bp, >Q30) corresponding to a coverage of 2938X.

**Table 1:** Capsella S-locus sequencing summary

**BAC Clone ID**	**SMRT Sequencing ID**	**Length of *S*-locus contig (bp)**	**Coverage SMRT raw assembly (x)**	**Number of SMRT reads**	**SMRT N50 read length (bp)**	**SMRT mean read length (bp)**	**Length of *S*-locus contig after trimming & circularisation (bp)**
CgrS-BAC1	pb_126-1	178,980	2690	56,575	19,187	11,836	156,636
CgrS-BAC1	pb_192-4	180,680	136	1,787	25,340	17,241	156,640
CgrS-BAC2	pb_274-14	164,087	160	1,421	28,120	20,433	153,560

We obtained one large contig containing the *S*-locus sequence for each of our three *S*-locus assemblies, with a length between 164 kbp and 178 kbp, as well as several smaller contigs containing parts of the *E. coli* genome or only cloning vector. Circularisation and vector trimming resulted in polished and trimmed assemblies of sequences containing complete *S*-locus sequences plus flanking regions of a total length of 156636, 156640 (for the two assemblies of CgrS-BAC1), and 153563 bp (for CgrS-BAC2), see Table 1.

Using SMRT sequencing allowed us to assemble the entire *S*-locus into one contig, in contrast to assemblies of the *S*-locus based on short-read data, which resulted in several contigs (Guo *et al.* 2011; Goubet *et al.* 2012; Durand *et al.* 2014). For short-read assemblies, even additional PCR-based measures to bridge the gaps between separate contigs often do not resolve the physical distances and relative orientations of genes for all haplotypes (Guo *et al.* 2011; Goubet *et al.* 2012). Quantifying variation in length, gene orientation and repeat content can be important in answering the question on reduced recombination at the *S*-locus (Goubet *et al.* 2012; Charlesworth 2016), but the diversity can only be fully revealed, if the *S*-haplotypes are assembled as continuous sequences.

### Assembly and sequencing errors

**Figure 1:**
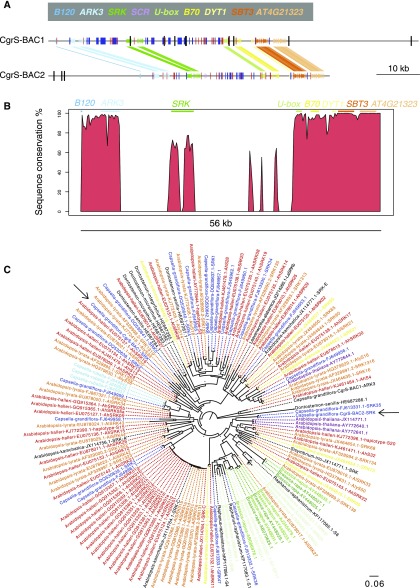
A *S*-locus sequence assemblies with two measures of indel errors indicated in black bars. Inference of indel errors are based on comparison of two independent SMRT-sequencing runs and assemblies of CgrS-BAC1 (upper) and alignment of Illumina short reads to assembly of CgrS-BAC2 (lower). Annotation of exons are shown as colored arrows, simple repeat sequences in red, and blue-boxes indicate positions of transposable elements. The genes flanking the *S*-locus are *ARK3* (light blue) and *U-box* (light green). *SCR* was only annotated in CgrS-BAC2. B *S*-locus sequence conservation between the two *Capsella S*-locus BACs, created by aligning the *S*-locus regions with LASTZ and comparing sequence homology (in % between 0 and 100) using a fixed window size of 250 bp. Sequence similarity between CgrS-BAC1 and CgrS-BAC2 drops steeply at the borders of the *S*-locus, corresponding to the genes *ARK3* and *U-box*, respectively, although some sequence similarity is also found at *SRK*. C ML phylogeny of all alignable *SRK* alleles (exon 1) above 500 bp from GenBank. Bootstrap support over 70% is represented with an asterisk (*). Our newly identified sequences, indicated with arrows, are found broadly distributed across the phylogeny.

There were no structural rearrangements present between the two *S*-locus contigs resulting from independent sequencing and assembly of two separate assemblies of CgrS-BAC1 (Figure 1A), suggesting that the rate of structural errors is low and these assemblies are accurate.

We report two measures for indel error rate. For CgrS-BAC1, indel errors were inferred by counting differences between two separate SMRT assemblies of the same BAC (Table 1., sequencing ID pb126_1 & pb192-4). For CgrS-BAC2, containing a different *S*-haplotype of *C. grandiflora*, indel errors were inferred by comparing MiSeq data to the SMRT assembly of the same BAC.

There were no SNP differences and 9 indel differences over an alignment length of 156,644 bp of the two assemblies of CgrS-BAC1 (Figure 1A). Thus, based on our technical replicates of library preparation and assembly, we estimate an indel error rate of 5.7×10^−5^ indels per bp with a ratio of single to double bp indels of 2:1. Notably, these indels were not specifically found in homopolymer regions.

Mapping of short-read Illumina MiSeq data to *S*-locus sequence CgrS-BAC2 resulted in an indel error rate estimate of 2.0×10^−5^ indels per bp over a sequence length of 153,563 bp (Figure 1A). Similarly, indels were the only errors and in this case all were single bp indels. Both methods for identifying indel error rates thus result in error rates on the order of 10^−5^ indels per bp, whereas no SNP errors were detected using either approach.

Current high throughput sequencing technologies show between 0.1 and ∼12% error rate of raw reads, with Illumina short read technologies generally below 1% and SMRT sequencing >10%, reviewed in (Reuter *et al.* 2015; Goodwin *et al.* 2016; Mardis 2017). The high error rate of SMRT sequencing raw reads is mitigated by a random distribution of these errors across individual reads and the ability to sequence circular fragments repeatedly, thus the consensus sequence is improved by multiple sequencing passes over the same continuous DNA molecule (Rhoads and Au 2015). With 15-fold coverage of single-molecule reads, the accuracy is raised to over 99% (Eid *et al.* 2009), but using the so called circular consensus reduces the average read length, weakening the keystone of long read sequencing (Travers *et al.* 2010; Hackl *et al.* 2014).

SMRT sequencing is useful for complete assembly of difficult loci (Bellec *et al.* 2016) or even genomes, microbial or chloroplast genomes have been assembled into fewer contigs than short read technologies, or even single continuous sequences were produced by SMRT sequencing alone, reviewed in (Rhoads and Au 2015). If one aims to study genetic variation at large divergent loci, SMRT-assemblies reveal complete genic and intergenic regions, but for higher resolution at the base-pair level, additional validation is necessary, as in a direct comparison of short and long read sequencing technology, SMRT-sequencing performs worse at the single-nucleotide variant calling (Quail *et al.* 2012). Also, indel errors in SMRT assemblies can cause frame-shifts and create difficulties for annotation via homology search (Du and Sun 2016) or could lead to false-positives in detection of frame-shift mutations.

At the order of 5.7 × 10^−5^ indels per bp our SMRT assembly already shows a lower error rate than error rates previously recorded for HGAP assemblies of SMRT sequences at: 99.9995% concordance with Sanger Sequences of microorganism genomes at ∼80-100 × coverage (Chin *et al.* 2013), though this study uses a higher coverage of 136 – 2690 x. Also, the assemblies performed better than error rates estimated for an *S*-locus study which found an average of 0.009 indel errors per bp (range 0–0.05), and an average of 0.02 substitutions errors per bp (range 0-0.1) based on 454 sequencing of *SRK* amplicons (Jørgensen *et al.* 2012).

The high accuracy even before error correction with short reads is likely owed to the fact that several Quiver polishing steps (see Materials and Methods) already work well at removing assembly errors if, as in our case, the coverage of long reads is high enough (Chin *et al.* 2013).

### Annotation of the S-locus

Annotation of our *S*-locus assemblies showed that this strategy resulted in full-length *S*-locus sequences (Figure 1A) containing both the *U-box* and *ARK3* flanking genes, as well as the key *S*-locus genes *SRK* and *SCR*. In CgrS-BAC1, *SCR* was not successfully annotated. The gene is known to be difficult to annotate due to its short nature and hyper variability. A phylogenetic tree of our *SRK* sequences and a set of publicly available *SRK* sequences confirms that our data falls within the range of sequence diversity observed at this locus in the Brassicaceae (Figure 1C). The sequence similarity drops steeply at the genes bordering the *S*-locus, *ARK3* and *U-box* (Figure 1B), and the only large region showing sequence conservation within the *S*-locus correspond to the gene *SRK*, a genetic determinant of self-incompatibility, as has also been found previously (Guo *et al.* 2011; Goubet *et al.* 2012).

### Cost and feasibility

Aligning short-read data to SMRT-assemblies for error correction eliminates the necessity of additional (PCR-based) validation, which enables a faster and simpler workflow, once the assembly and error correction is complete. SMRT sequencing is still relatively costly, adding to the costs of BAC library production (∼1700 € at time of publishing), but for certain studies long reads are indispensable, for instance to assemble regions of high repeat content and to accurately assemble intergenic regions (Rhoads and Au 2015). Using a double platform approach takes more financial resources, time and data processing, but can generate assemblies of higher accuracy than SMRT sequencing alone (Rhoads and Au 2015).

High quality assemblies are necessary for many genetic studies, by the alignment of short read data directly to SMRT long reads, hybrid software are able to improve the accuracy of SMRT sequencing long reads (Au *et al.* 2012; Koren *et al.* 2012; Hackl *et al.* 2014; Salmela and Rivals 2014), *e.g.*, PBcR from ∼85% up to 99.9% (Koren *et al.* 2012), which can then be *de novo* assembled with higher confidence. Hybrid assemblies however are computationally intensive, especially early programs (Au *et al.* 2012; Koren *et al.* 2012; Salmela and Rivals 2014), as they must allow for more mismatches between short and long reads than other assembly methods. The approach of using short reads to error correct SMRT assemblies is a computationally simpler and efficient way to generate highly accurate assemblies.

### Conclusions

We show that SMRT sequencing of BACs is an efficient way to obtain high-quality assemblies of the Brassicaceae *S*-locus, a locus that has been difficult to study due to its high content of repeats and high divergence among alleles. Independent SMRT sequencing runs of the same BAC clone allow us to estimate an error rate of 5.7 × 10^−5^ indels per bp. These errors can efficiently be corrected using short reads, and such correction is important especially in the context of highly accurate studies of functional gene variants.

This approach can be useful for studies of other genomic regions characterized by high divergence and repetitive content, such as other loci under long-term balancing selection (Fijarczyk and Babik 2015), where reference based short-read sequencing technologies are not feasible.

## Supplementary Material

Supplemental material is available online at www.g3journal.org/lookup/suppl/doi:10.1534/g3.117.300467/-/DC1.
