# Supplementary material for: Targeted Long-Read Sequencing of a Locus Under Long-Term Balancing Selection in Capsella
Source: G3 (Bethesda). 2018 Feb 20;8(4):1327–33. doi: 10.1534/g3.117.300467 (PMC5873921; doi:10.1534/g3.117.300467)
Supplement: Supplementary file 1 [file 1327TableS1.docx]

**Table S1**. *SRK* sequences used in this study (Genbank accession numbers)

| Species | Accession number | |  |  |  |  |  |  |
| --- | --- | --- | --- | --- | --- | --- | --- | --- |
| *Arabidopsis arenosa* | JX464654.1 | JX464627.1 | JX464615.1 | JX464623.1 | JX464618.1 | JX464616.1 | |  |
| *Arabidopsis halleri* | JX114764.1 | AY772644.1 | KJ772390.1 | KJ772396.1 | GQ915357.1 | GQ915356.1 | GQ915355.1 | GQ915360.1 |
|  | GQ915354.1 | GQ915358.1 | GQ915353.1 | GQ915351.1 | GQ915352.1 | GQ915359.1 | GQ915359.1 | GQ915363.1 |
|  | GQ915361.1 | KJ461471.1 | KJ461478.1 | KJ461479.1 | KJ461484.1 | EU878014.1 | EU878015.1 | EU878012.1 |
|  | EU075125.1 | EU075136.1 | EU075138.1 | EU075138.1 | EU075131.1 | EU075132.1 | EU075133.1 | EU075134.1 |
|  | GQ915364.1 | GQ915365.1 | EU075143.1 | EU075140.1 | EU878009.1 | EU075130.1 | EU075124.1 | EU075135.1 |
|  | EU878011.1 | EU878010.1 | EU075127.1 | EU075142.1 | EU075137.1 GQ915361.1 | |  |  |
| *Arabidopsis kamchatica* | JX114764.1 | JX114771.1 | JX114756.1 | |  |  |  |  |
| *Arabidopsis lyrata* | GQ915366.1 | GQ915366.1 | GQ915359.1 | HQ379629.1 | KJ772405.1 | KJ772408.1 | KJ772418.1 | HQ379630.1 |
|  | HQ379631.1 | AF328993.1 | AF328999.2 | AF328996.2 | AF328997.2 | AF328990.2 | AF328992.2 | AF328994.2 |
|  | GQ915369.1 | GQ915370.1 | AF329000.2 | AF328995.1 | AF328998.2 | FJ867321.1 | AF328991.2 | JX464650.1 |
|  | JX464654.1 | EU878023.1 | EU878024.1 | EU878025.1 | JX464639.1 | EU878016.1 | EU878019.1 | EU878021.1 |
|  | EU878020.1 | JX464644.1 | EU878017.1 | |  |  |  |  |
| *Arabidopsis thaliana* | JX114771.1 | AY772640.1 | AY772641.1 | AY772644.1 |  | |  |  |
| *Brassica napus* | AB086976.1 | AJ245479.1 | AY448031.1 | AY448033.1 | AY448035.1 | |  |  |
| *Brassica oleracea* | JX114771.1 | AJ306587.1 | JX861859.1 | GQ915361.1 | AB070624.1 | AJ306591.1 | AJ306584.1 | |
| *Brassica rapa* | AB070625.1 | AY448029.1 | AY448025.1 | AY448027.1 | |  |  |  |
| *Capsella grandiflora* | DQ530638.1 | DQ530642.1 | DQ530637.1 | FJ613333.1 | DQ530640.1 | DQ530639.1 | FJ613332.1 | FJ613331.1 |
|  | DQ530641.1 | FJ649961.1 | FJ649962.1 | FJ649959.1 | FJ649957.1 | FJ649956.1 | FJ649955.1 | FJ649953.1 |
|  | FJ613330.1 | FJ649960.1 | FJ649958.1 | |  |  |  |  |
| *Capsella rubella* | FJ649926.1 | FJ649937.1 | FJ649942.1 | |  |  |  |  |
| *Dontostemon integrifolius* | HE687285.1 | |  |  |  |  |  |  |
| *Dontostemon micranthus* | HE687283.1 | HE687282.1 | |  |  |  |  |  |
| *Dontostemon senilis* | HE687286.1 | HE687287.1 | HE687284.1 | |  |  |  |  |
| *Leavenworthia alabamica* | JQ714260.1 | |  |  |  |  |  |  |
| *Raphanus raphanistrum* | KP117083.1 | KP117085.1 | hKP117084.1 | |  |  |  |  |
| *Sisymbrium irio* | JX114771.1 | |  |  |  |  |  |  |
